# Emotional distress and risk of dental caries: Evaluating effect modification by chronic conditions among low-income African American caregivers in Detroit, Michigan

**DOI:** 10.3389/fpubh.2023.1050511

**Published:** 2023-01-20

**Authors:** Sungwoo Lim, Marisol Tellez, Amid I. Ismail

**Affiliations:** Kornberg School of Dentistry, Temple University, Philadelphia, PA, United States

**Keywords:** dental caries, emotional distress, chronic disease, oral hygiene, sugar consumption

## Abstract

**Background/aim:**

Limited research has been conducted regarding the association between mental illness and dental caries. We studied the impact of emotional distress on current and new dental caries among low-income African-American caregivers in Detroit, Michigan and if this association was mediated by poor oral hygiene and sugar consumption and modified by a chronic health condition.

**Methods:**

Data came from Detroit Dental Health Project, a prospective cohort study of low-income African American caregivers and their children. We focused on baseline (*n* = 1,021) and 4-year follow-up participants (*n* = 614). Dental caries were assessed using the International Caries Detection and Assessment System. The study outcomes included two baseline caries outcomes (counts of non-cavitated lesions, baseline counts of cavitated lesions) and two outcomes of new caries over 4 years (new cavitated lesions and new non-cavitated lesions). The exposure was emotional distress. We performed multivariable quasi-Poisson regression analysis to test the association between emotional distress and caries. We tested effect modification by stratifying data by chronic health conditions and performed causal mediation analysis to test an indirect effect of oral hygiene and sugar consumption.

**Results:**

Ninety six percent of the caregivers were female, and their average age was 28 years old. Thirteen percent reported emotional distress at baseline. After accounting for potential confounding, emotional distress was positively associated with cavitated lesions at baseline (IRR = 1.36, 95% CI = 1.08, 1.70). Among those with a chronic health condition, stronger association was observed (IRR = 1.73, 95% CI = 1.27, 2.35). After 4 years, those with emotional distress and chronic health conditions had an increased risk of developing non-cavitated carious lesions (IRR = 1.41, 95% CI = 1.06, 1.88). Poor oral hygiene explained 51% of the association between emotional distress and baseline cavitated lesions (natural indirect effect = 1.16, 95% CI = 1.02, 1.33), but there was no evidence for an indirect effect of sugar consumption.

**Conclusion:**

In this group of young, African-American caregivers with low socioeconomic status, dental caries was associated with emotional distress. This association was explained by poor oral hygiene and strengthened among those who reported a chronic health condition.

## Introduction

High burden of dental caries has been observed among people with mental illness (1). Depressive symptoms were significantly associated with dental caries among pregnant women from the Northern Appalachia, United States (2). According to a Korean study, adults with life-time depression were less likely to brush their teeth or make oral health visits despite having oral health problems, and more likely to experience various oral health problems such as toothache (3). Along with poor oral hygiene practices, individuals with mental illness may be more likely to consume a higher amount of sugars with higher frequencies or smoke as a coping mechanism (4, 5), which are positively linked to dental caries risk. A potential pathway by which mental illness and dental caries are associated may be explained by intake of psychotropic medication (6). Antidepressants are known to decrease salivary flow rate and increase the risk of dental caries.

Similar to mental illness, the association between a chronic health condition and dental caries has been observed in several cross-sectional studies (7–9). In a Korean study, women who were overweight or obese or elderly were more likely to develop advanced caries compared with those who were underweight or normal weight (7). A recent scoping review of the existing studies examined the relationship between chronic diseases and dental outcomes, and concluded that dental caries was associated with metabolic diseases (8). Another study of individuals from Saudi Arabia found that the reduction of salivary pH and oral microbiome changes could increase among those with Type 1 diabetes (9). Lastly, a recent study of 1,294 migrants in Europe analyzed self-reported cross-sectional data and found that mental and general health status was positively associated with dental health conditions (10).

Despite evidence that dental caries is associated with mental illness and chronic health conditions, however, limited research has been conducted to describe and understand the role of chronic conditions in the relationship between mental illness and dental caries using clinical and longitudinal data. To address the current gap in the literature, we used population representative, longitudinal data from low-income African-American caregivers in Detroit, Michigan, and aimed to test (1) the association between emotional distress and dental caries, and (2) the potential effect modification by a chronic health condition. Given that co-occurrence of mental and physical health conditions is highly prevalent, we postulate that the association between mental illness and dental caries is stronger among those with a chronic health condition. In addition, to understand how emotional distress affects dental caries development, we tested whether poor oral hygiene, measured by disclosing old and new plaque, and sugar consumption could mediate the relationship between emotional distress and dental caries.

## Materials and methods

### Study population

Data for this study came from The Detroit Dental Health Project, a 4 year prospective cohort study to investigate the oral health determinants of low-income African-American children and their caregivers from Detroit, Michigan. A stratified 2-stage area probability sampling was used to create a representative sample of low-income African-American households that comprised of at least one caregiver of a child ages 0 to 5 years. In 2001-02 (baseline), 1,021 child-caregiver pairs participated in the dental examination and survey interviews. They were followed up in 2004-05 as well as 2007. Details of the sampling and data collection procedures have been described in the previous report (11). In this study, we focused on caregivers who participated in the baseline survey and dental examination (*n* = 1,021) and the second follow-up after 4 years (*n* = 614).

The study protocol was approved by the Institutional Review Board for Health Sciences at the University of Michigan and caregivers of all participants gave written consent for inclusion in this study (IRB# 1325).

### Measures

Dental caries was assessed by dentists using the International Caries Detection and Assessment System (ICDAS). The study outcomes included baseline counts of non-cavitated lesions (ICDAS scores: 01, 02) and baseline counts of cavitated lesions (ICDAS scores: 03, 04, 05, 06). In addition, by comparing baseline data with follow-up data, we calculated counts of new non-cavitated lesions and new cavitated lesions over 4 years, and used them as additional outcomes. The exposure variable was emotional distress at baseline, which was measured by asking the question “during the past 1 week, how much have you been bothered by emotional problems (such as feeling anxious, depressed or irritable)?” Those who responded “quite a lot” or “extremely” were considered to have emotional distress. A chronic health condition was determined if caregivers responded “yes” to one of the following questions: “Has a doctor ever diagnosed you with a heart murmur or a valve problem, an infection in the lining of your heart called endocarditis, diabetes, epilepsy or another seizure disorder, or AIDS/HIV?” Those who reported having a heart, kidney, or other organ transplant, a heart attack or chest pain, or a surgically implanted heart valve, stent, shunt, or an artificial joint were also considered to have a chronic health condition. Oral hygiene was measured using the patient hygiene performance index. We identified old and new debris on 60 tooth surfaces from 6 teeth (maxillary right first molar, maxillary right central incisor, maxillary left first molar, mandibular left first molar, mandibular left central incisor, mandibular right fist molar), and created two oral hygiene measures: counts of tooth surfaces with old plaque, and counts of tooth surfaces with new plaque (12). Dietary data were collected by trained interviewers using the Block 98.2 Food Frequency Questionnaire. Daily sugar consumption in grams was then estimated using Block's algorithm based on data from several USDA National Food Consumption Surveys. To account for potential confounding, we included caregivers' demographic characteristics (age, sex, household income, education attainment, full-time employment, number of children), dental insurance, and brushing frequencies were collected *via* self-reported responses to survey questions.

### Statistical analysis

First, we described baseline demographic characteristics, chronic health conditions, and dental caries of caregivers overall as well as by emotional distress. Difference in baseline characteristics by emotional distress was tested using chi-squared tests or independent *t*-tests. We then conducted multivariable quasi-Poisson regression analysis to test whether emotional distress was associated with baseline and new counts of non-cavitated and cavitated lesions after accounting for potential confounding. To test effect modification by a chronic health condition, we stratified data by a chronic health condition and re-ran the multivariable regression analysis. Lastly, to test whether oral hygiene and/or sugar consumption mediate the relationship between emotional distress and dental caries, we performed causal mediation analysis. Specifically, we estimated mediation weights (i.e., inverse of probability of a mediator value) by using oral hygiene or sugar consumption as an outcome and the same set of covariates from the main multivariable regression analysis except for brushing frequencies. These weights were multiplied with survey weights and incorporated into the complex sample design. Natural direct and indirect effects were estimated after accounting for complex sample design, and percent mediated for oral hygiene and sugar consumption was estimated using the formula from VanderWeele and Vansteelandt (13). Statistical analyses were conducted using R software 3.5.2 (Vienna, Austria). Complex sample design and survey weights were accounted for. For complex sample data analysis, we used R survey package (14) and for causal mediation analysis, we used Medflex package (15). Statistical significance was determined using two-sided *p* < 0.05.

## Results

Table 1 shows that 96% of the caregivers were female. The average age of the caregivers at baseline was 28 years old. Reflecting the target population and area, about half did not have high school degrees and reported their annual household income <$10,000. The average brushing frequency was 9 times per week, and 76% had dental insurance. Moreover, 39% reported having a chronic health condition, and 13% reported emotional distress at baseline. None of baseline characteristics except for a chronic health condition was statistically significantly different between those with and without emotional distress. For those with emotional distress, 51% reported a chronic health condition, which was significantly higher than those without emotional distress (36%).

**Table 1:** Selected characteristics of African-American caregivers, Detroit, Michigan, 2002–2003.

	**Total**	**Emotional distress** **Yes**	**Emotional distress** **No**
N	1,021	151	870
Mean age in years (standard error)	29.0 (0.3)	29.3 (0.8)	28.9 (0.3)
Sex			
Female	96%	97%	96%
Male	4%	3%	4%
Mean number of children (standard error)	1.7 (0.03)	1.6 (0.1)	1.8 (0.04)
Annual household income			
< $10,000	44%	49%	44%
$10,000–$19,999	43%	41%	43%
$20,000 or higher	13%	10%	13%
Highest attained education			
< High school	38%	32%	39%
High school diploma or higher	62%	68%	61%
Full-time employment	29%	21%	30%
Dental insurance	76%	77%	76%
Mean brushing frequency in the past 1 week (standard error)	9.4 (0.2)	9.1 (0.5)	9.5 (0.2)
Chronic health condition	39%	**51%**	**36%**

Numbers in bold indicate statistical significance at p < 0.05.

The mean number of baseline non-cavitated lesions and cavitated lesions was 18.7 (SE = 0.4) and 8.7 (SE = 0.6), respectively (Table 2). Over 4 years, the caregivers developed 12.8 (SE = 0.6) new non-cavitated and 5.4 (SE = 0.4) new cavitated lesions on average. These dental caries outcomes were compared by caregivers' emotional distress status, and no substantial difference was observed. The independent *t*-test confirmed the null finding. When data were restricted to the caregivers with a chronic health condition, similar trends were observed except for significantly higher numbers of baseline cavitated lesions among those with emotional distress (13.5 vs. 8.1). The mean number of tooth surfaces with old plaque and new plaque were 6.9 (SE = 0.3) and 25.6 (SE = 0.4), respectively, and similar between those with and without emotional distress. The mean daily sugar consumption was 226 grams for all caregivers. Statistically significantly higher sugar consumption was observed among those with emotional distress vs. those without emotional distress s (252 vs. 222 g). When restricted to caregivers with chronic health conditions, higher sugar consumption was observed among those with emotional distress (258 vs. 212 g).

**Table 2:** Dental caries and oral hygiene at baseline and follow-up among African-American caregivers, Detroit, Michigan, 2002–2007.

				**Chronic health condition**	
	**Total**	**Emotional distress: Yes**	**Emotional distress: No**	**Emotional distress: Yes**	**Emotional distress: No**
Dental caries					
Non-cavitated lesions at baseline	18.7 (0.4)	17.8 (0.7)	18.9 (0.5)	17.6 (1.2)	18.1 (0.6)
Cavitated lesions at baseline	8.7 (0.6)	10.9 (1.6)	8.4 (0.6)	**13.5 (2.8)**	**8.1 (1.0)**
Non-cavitated lesions at follow-up	12.8 (0.6)	13.0 (1.5)	12.7 (0.6)	15.4 (2.8)	11.0 (1.1)
Cavitated lesions at follow-up	5.4 (0.4)	5.0 (0.6)	5.5 (0.4)	5.3 (0.8)	4.7 (0.6)
Oral hygiene					
Old plaque at baseline	6.9 (0.3)	6.7 (0.7)	6.9 (0.3)	7.1 (1.0)	7.3 (0.6)
New plaque at baseline	25.6 (0.4)	25.9 (0.9)	25.5 (0.4)	25.8 (1.6)	24.5 (0.7)
Total sugar consumption (in grams) at baseline	225.7 (4.3)	251.5 (10.9)	221.5 (4.4)	257.8 (17.3)	212.1 (7.8)

Numbers in bold indicate statistical significance at *p* < 0.05.

After accounting for potential confounding, emotional distress was positively associated with cavitated lesions at baseline (Table 3; IRR = 1.36, 95% CI = 1.08, 1.70). When stratified by a chronic health condition, a stronger association was observed among those with a chronic health condition (IRR = 1.73, 95% CI = 1.27, 2.35). On the other hand, emotional distress was not associated with baseline non-cavitated lesions for all caregivers as well as those with a chronic health condition. After 4 years, the caregivers with emotional distress at baseline were associated with increased risk of development of non-cavitated lesions only among those with chronic health conditions (IRR = 1.41, 95% CI = 1.06, 1.88), while emotional distress was not associated with risk of developing cavitated lesions.

**Table 3:** Incidence rate ratio for dental caries by emotional distress from the multivariable regression analysis among African-American caregivers, Detroit, Michigan, 2002–2007.

	**Model for baseline non-cavitated lesions**	**Model for baseline cavitated lesions**	**Model for new non-cavitated lesions**	**Model for new cavitated lesions**
	IRR (95% CI)	IRR (95% CI)	IRR (95% CI)	IRR (95% CI)
Emotional distress: yes vs. no	0.97 (0.88, 1.06)	1.36 (1.08, 1.70)	1.07 (0.89, 1.29)	1.00 (0.77, 1.29)
For those w/chronic disease				
Emotional distress: yes vs. no	0.99 (0.86, 1.13)	1.73 (1.27, 2.35)	1.41 (1.06, 1.88)	1.12 (0.75, 1.68)

According to the causal mediation analysis (Table 4), counts of old debris significantly mediated the relationship between emotional distress and baseline number of cavitated lesions (natural indirect effect = 1.16, 95% CI = 1.02, 1.33). Emotional distress was also significantly associated with baseline cavitated lesions when an indirect pathway *via* oral hygiene were controlled (natural direct effect = 1.19, 95% CI = 1.05, 1.34). The estimated percent of the association between emotional distress and caries mediated by poor oral hygiene were 51%. On the other hand, there was no evidence for mediation by number of new plaque and sugar consumption. Further, data did not support a mediation role of oral hygiene and sugar consumption for the association between emotional distress and non-cavitated lesions (data not shown).

**Table 4:** Causal mediation analysis of oral hygiene and sugar consumption on the association between emotional distress and cavitated lesions at baseline.

	**Natural direct effect IRR (95% CI)**	**Natural indirect effect IRR (95% CI)**	**Percent mediated**
Old plaque	1.16 (1.02, 1.33)	1.19 (1.05, 1.34)	51%
New plaque	1.31 (1.16, 1.47)	1.00 (0.89, 1.12)	0%
Sugar consumption	1.27 (1.13, 1.43)	1.03 (0.92, 1.15)	12%

## Discussion

In this study of low-income African American caregivers of young children, we found that emotional distress was associated with having higher numbers of cavitated lesions. We further found a significant effect modification by chronic health conditions. The expected increase of caries lesions among caregivers with emotional distress, compared with those without emotional distress, increased from 36 to 73% when restricted to those with a chronic health condition. Emotional distress was also linked to a higher risk of developing new non-cavitated carious lesions in 4 years among caregivers with chronic health conditions.

The positive relationship between emotional distress and dental caries in this study is consistent with a previous study of women from Northern Appalachia that reported higher mean DMFT among those with depressive symptoms (2). Our study contributes to strengthening the existing evidence by demonstrating that the positive relationship between mental health and caries remained true after accounting for potential confounding.

The significant mediation effect by old plaque suggests that caregivers with emotional distress might neglect proper oral hygiene practices for long periods, which in turn increases the risk of developing dental caries. This indirect pathway has been partially supported by the previous findings that poor oral health care, measured by accumulation of plaque, was quite prevalent among individuals who received psychiatric outpatient cares in Sweden (16), and was associated with suicidal ideation/attempt among Nigerian adolescences (17). Given that about half of the association between emotional distress and dental caries was explained by poor oral hygiene, programs or support for maintaining and promoting good oral hygiene *via* tailored dental education should be considered to reduce caries (18).

By testing an effect modification by a chronic health condition, we were able to identify the caregivers with higher risk of developing caries attributed to emotional distress beyond individual and environmental risk factors. Burdened with co-occurrence of mental and physical illnesses, they might be less motivated to practice proper oral hygiene and more likely to consume foods with high sugar content than the other caregivers, as shown by a significantly higher sugar consumption associated with emotional distress in this study. Given a well-established etiological relationship between sugar consumption or poor oral hygiene and dental caries (19, 20), these oral health and dietary behaviors might explain increased risk of caries among the caregivers with both emotional distress and a chronic health condition. However, we could not find evidence on the mediation *via* poor oral hygiene and sugar consumption. Future studies are warranted to explore and test an underlying mechanism that explains an effect modification by a chronic health condition.

A main strength of the current study was the use of longitudinal dental caries data that were calibrated and examined by dentists *via* ICDAS (21). In addition, the study used population representative data from the low-income African American caregivers living in deprived urban areas, which allowed us to assess a contribution of mental illness to dental caries and a role of a chronic health condition while effectively controlling for neighborhood-level confounding.

However, this study had several limitations. First, the participants could have over or under-reported their status of emotional distress due to recall error or social desirability. Second, although caregivers were sampled from the well-defined geographic area with similar individual and environmental characteristics, bias due to unobserved confounding could not be ruled out. Third, dietary behavior data such as snacking patterns were not collected, which might explain limited evidence of indirect effect of sugar consumption.

## Conclusions

In conclusion, emotional distress was associated with severity of dental caries at baseline among African American caregivers of young children from the low-income urban area. As expected, additional burden due to a chronic health condition strengthened this association, and among the caregivers with a chronic health condition, emotional distress was associated with higher numbers of new non-cavitated lesions in 4 years. This finding suggests that resources for dental caries prevention should be prioritized to individuals with both emotional distress and chronic conditions. Given a large mediation effect by poor oral hygiene, promoting proper oral hygiene practices should be considered as an effective intervention to reduce the risk of dental caries.

## Data availability statement

The raw data supporting the conclusions of this article will be made available by the authors, without undue reservation.

## Ethics statement

The studies involving human participants were reviewed and approved by the Institutional Review Board for Health Sciences at the University of Michigan. The patients/participants provided their written informed consent to participate in this study.

## Author contributions

SL conceptualized the study, led the writing of the article, and statistical analysis. MT and AI led the project, including the study design, sample selection, data collection, assisted with the interpretation of data, and policy implication. All authors contributed to the review and editing of drafts of the article and approved the version to be published.
